# Cholera Toxin Encapsulated within Several *Vibrio cholerae* O1 Serotype Inaba Outer Membrane Vesicles Lacks a Functional B-Subunit

**DOI:** 10.3390/toxins11040207

**Published:** 2019-04-06

**Authors:** Elnaz S. Rasti, Angela C. Brown

**Affiliations:** Department of Chemical and Biomolecular Engineering, Lehigh University, Bethlehem, PA 18015, USA; elr314@lehigh.edu

**Keywords:** *Vibrio cholerae*, cholera toxin, outer membrane vesicles, GM1 ganglioside, Type II secretion system

## Abstract

Cholera toxin (CT), the major virulence factor of *Vibrio cholerae*, is an AB5 toxin secreted through the type II secretion system (T2SS). Upon secretion, the toxin initiates endocytosis through the interaction of the B pentamer with the GM1 ganglioside receptor on small intestinal cells. In addition to the release of CT in the free form, the bacteria secrete CT in association with outer membrane vesicles (OMVs). Previously, we demonstrated that strain 569B releases OMVs that encapsulate CT and which interact with host cells in a GM1-independent mechanism. Here, we have demonstrated that OMV-encapsulated CT, while biologically active, does not exist in an AB5 form; rather, the OMVs encapsulate two enzymatic A-subunit (CTA) polypeptides. We further investigated the assembly and secretion of the periplasmic CT and found that a major fraction of periplasmic CTA does not participate in the CT assembly process and instead is continuously encapsulated within the OMVs. Additionally, we found that the encapsulation of CTA fragments in OMVs is conserved among several Inaba O1 strains. We further found that under conditions in which the amount of extracellularly secreted CT increases, the concentration of OMV-encapsulated likewise CTA increases. These results point to a secondary mechanism for the secretion of biologically active CT that does not depend on the CTB-GM1 interaction for endocytosis.

## 1. Introduction

Cholera is an acute and often fatal diarrheal disease that is caused by effective colonization of *Vibrio cholerae* in the small intestine [1,2,3]. More than 200 serogroups of *V. cholerae* have been identified so far. However, only the O1 and O139 serogroups, which can express cholera toxin (CT), the key virulence factor of *V. cholerae*, are able to cause epidemic cholera. *V. cholerae* O1 strains are classified into two biotypes, El Tor and Classical [4]. CT is an 84 kDa protein composed of an enzymatic A-subunit (CTA), which is non-covalently linked to a pentamer of B-subunits (CTB5). The A-subunit (28 kDa), initially synthesized as a single polypeptide, expresses activity after a proteolytic cleavage, also called “nicking,” at residue Arg-192, producing two functional A1 (∼21.8 kDa) and A2 (∼5.4 kDa) domains, which remain covalently associated by a disulfide bond. CTA1 is responsible for the ADP-ribosyltransferase activity, and the alpha-helical CTA2 tethers the CTA1 and CTB5 subunits together [5,6,7,8].

The CT holotoxin is secreted via the two-step type II secretion system (T2SS). First, CT subunits are synthesized as precursors and are translocated across the inner membrane via the Sec machinery to the periplasmic space, where the subunits fold and spontaneously assemble into the holotoxin. Second, the extracellular protein secretion (Eps) operon recognizes the pentameric CTB5, which carries the secretion signal, and exports CT across the outer membrane to the extracellular medium [9,10]. Upon secretion, CTB binds to its receptor, ganglioside GM1 (GM1), on the host cell surface, which facilitates the endocytosis of the CT-GM1 complex [11]. The complex then traverses in a retrograde fashion through the trans-Golgi network into the endoplasmic reticulum (ER) [12]. The ER chaperone, protein disulfide isomerase (PDI), dissociates CTA1 from the rest of the toxin, leading to the spontaneous unfolding of the subunit. The unfolded CTA1 then triggers the ER-associated degradation (ERAD) machinery to retro-translocate to the cytosol, where it activates adenylate cyclase, which in turn increases the cyclic AMP (cAMP) level, causing activation of the cystic fibrosis transmembrane conductance regulator (CFTR). This leads to an enhanced efflux of chloride ions and water into the intestinal lumen, inducing watery diarrhea [13,14,15,16].

In addition to the well-studied soluble form of CT, the toxin can also be found in association with outer membrane vesicles (OMVs) in a physiologically active form [17,18]. OMVs are nanometer-scale, spherical particles secreted from the outer membrane of Gram-negative bacteria. These vesicles comprise various bacterial components including lipopolysaccharide (LPS), outer membrane proteins (OMPs), phospholipids, periplasmic proteins, DNA, and RNA. OMVs play a major role in the pathogenicity of Gram-negative bacteria by transporting a complex of biologically active virulence factors to their target cell [19,20].

It has been reported that the NaCl level in the culture medium has a biphasic impact on CT production in Classical strains of *V. cholerae*, with NaCl concentrations below 50 mM inhibiting production of the toxin [21,22]. However, in our previous study on the *V. cholerae* 569B strain (Classical), we demonstrated that under a low-salt growth condition, the majority of the secreted CT is in an OMV-associated form and is located exclusively inside the vesicles. This location prevents the toxin from being detected by conventional CT-detection assays. Unlike soluble CT, which employs GM1 to initiate cellular internalization, we found that under this low-salt condition, the physiologically active OMV-encapsulated CT is unable to bind GM1 on the host cell surface. Therefore, the OMV-associated CT is trafficked to the host cells in a GM1-independent mechanism [18]. We hypothesized that because the B-subunit mediates the interaction of the holotoxin with GM1, the OMV-encapsulated form of the toxin might exclude the B-subunit. Here, we continued our studies with the *V. cholerae* 569B strain cultured in a nutrient broth medium under a range of osmolarities to investigate the association of specific CT subunits with OMVs and further characterize this important delivery system. Additionally, we assessed the strain-dependence of our results by analyzing OMVs from two pandemic O1 El Tor strains, C6706 and N16961.

## 2. Results

### 2.1. The Effect of Osmolarity of the Growth Medium on Association of CT with 569B OMVs

Previous studies on *V. cholerae* Classical strains have demonstrated that secretion of CT is regulated by the level of NaCl in growth medium and is inhibited under low-salt condition [21,22]. Using a GM1 ELISA assay, Gardel et al. [22] have demonstrated that the optimal NaCl concentration for total CT secretion in various Classical strains is in the range of 60–100 mM. On the other hand, our previous studies on Classical *V. cholerae* 569B demonstrated that CT is also secreted under low-salt conditions; however, because a majority of the toxin is encapsulated within the OMVs and is not located on the surface of the vesicles, it is not detectable with the GM1 ELISA. To initiate this study, we first wanted to investigate whether a change in culture osmolarity influences the association of extracellularly secreted CT with the surface of OMVs.

**Figure 1:**
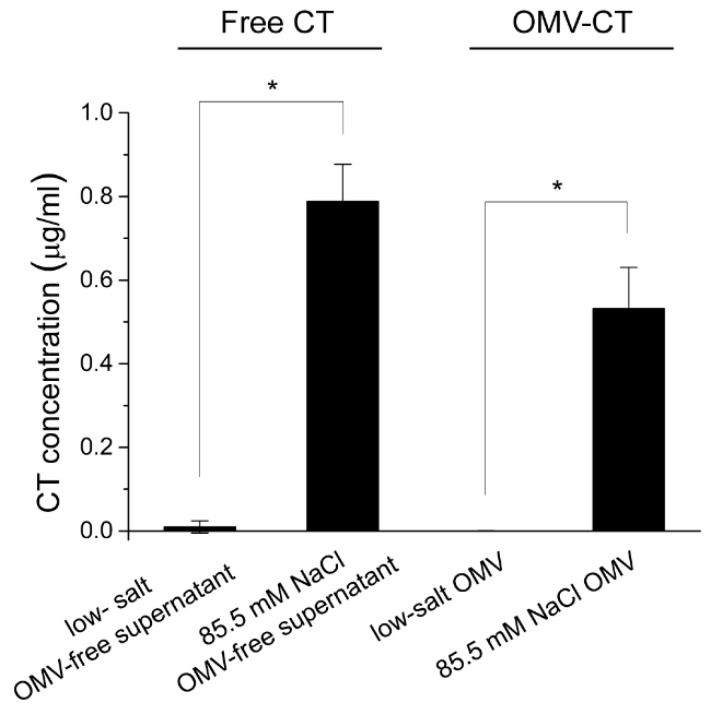
Effect of medium osmolarity on association of cholera toxin (CT) with outer membrane vesicles (OMVs). A ganglioside GM1 (GM1) ELISA was performed for OMVs and OMV-free supernatant fractions purified from either low-salt or 85.5 mM NaCl-containing 569B growth medium. The increase in salt concentration in the growth medium increased the secretion of CT in the free form (OMV-free supernatant), as well as on the surface of the OMVs. Data are shown as mean ± SD (*n* = 3). The level of significance was determined using one-way analysis of variance followed by Bonferroni post-hoc test: 2 comparisons; * *p* < 0.05/2 = 0.025.

Therefore, *V. cholerae* 569B was grown to an OD_600_ of 1.0 in a nutrient broth containing 85.5 mM NaCl. Another sample was also cultured in standard nutrient broth with no added salt and used as a control. OMVs and OMV-free supernatant fractions were then collected from each sample. A GM1 ELISA assay was performed to quantify the CT concentration in each sample, using a serial dilution of CT as a standard and an anti-CT polyclonal antibody. As shown in Figure 1, under the low-salt culture condition, no signal was detected for CT on the surface of OMVs, consistent with our previous results [18], and a low concentration of CT was found in the OMV-free supernatant fraction, consistent with reported results [22]. As expected, under the NaCl-rich condition, the concentration of CT in the OMV-free supernatant fraction increased significantly. Correspondingly, the concentration of CT on the surface of the OMVs increased, comprising approximately 40% of the total secreted CT. This result indicates that under a low-salt growth condition, no CT is found on the surface of the OMVs, but under a salt-rich growth condition, a large fraction of secreted CT is associated with the OMV surface.

### 2.2. OMVs Isolated from Low-Salt V. cholerae Culture Do Not Carry Intact CT Holotoxin

Although CT does not bind to the surface of OMVs under low-salt culture conditions, we have previously demonstrated that CT is encapsulated within the OMVs under this condition. Our results in Figure 1 shows that under salt-rich conditions, the CT on the surface of OMVs binds to the GM1 receptor. However, our previous results indicated that OMV-encapsulated CT does not require GM1 for host cell intoxication [18], suggesting that the encapsulated CT may not be holotoxin. Hence, we sought to characterize the structure of the OMV-encapsulated CT. To specifically characterize the lumenal CT and for the sake of simplicity in the analysis of this form of the toxin, we performed the rest of our studies under low-salt conditions, unless stated otherwise.

**Figure 2:**
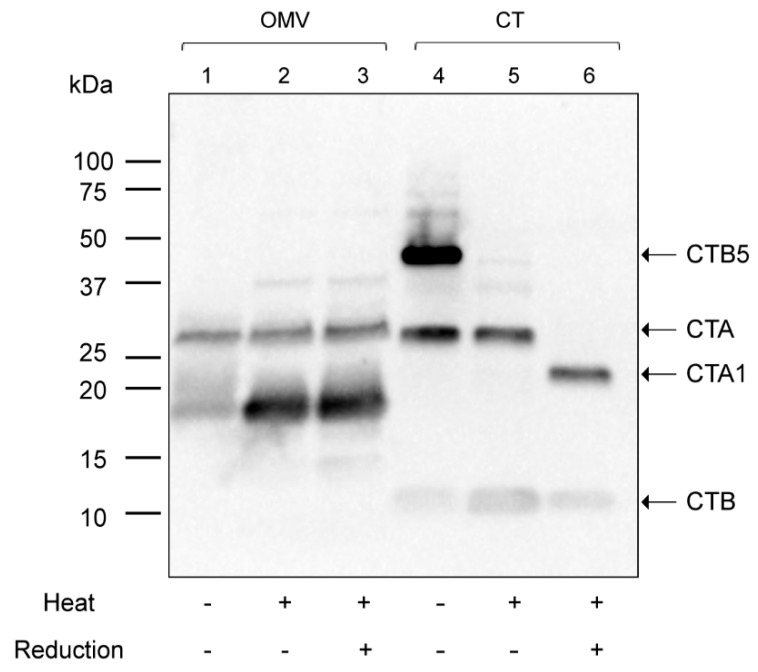
Western blot analysis of 569B OMVs and purified CT. Lanes 1 and 4: 569B OMVs and CT under non-boiled and non-reduced conditions. Lanes 2 and 5: 569B OMVs and CT under boiled and non-reduced conditions. Lanes 3 and 6: 569B OMVs and CT under boiled and reduced conditions. Two non-reducible anti-CT antibody-reactive bands were detected in 569B OMVs in each condition, but no band corresponding to the CTB5 pentamer was detected in the OMV sample.

For this reason, we first isolated OMVs from a low-salt nutrient broth culture to compare the OMV-encapsulated CT structure to that of purified CT using Western blot under (i) non-boiled and non-reduced, (ii) boiled and non-reduced, or (iii) boiled and reduced conditions and using a polyclonal anti-CT antibody. Under the non-boiled and non-reduced conditions, the CT sample migrated as a CTB5 pentamer with an apparent molecular weight of 45 kDa, a CTA-subunit (28 kDa), and a CTB monomer (11.6 kDa), as shown in Figure 2, lane 4. Surprisingly, no band was found corresponding to the CTB5 pentamer in the OMVs under these conditions (lane 1). Instead, only a band that migrated in a similar manner as the CTA band (28 kDa) and an unknown band with a molecular weight of approximately 18 kDa were detected.

Under the boiled and non-reduced conditions, the CT sample dissociated into a CTB monomer and a CTA-subunit, as shown in lane 5. In the corresponding OMV sample, the same two bands detected in lane 1 (18 and 28 kDa) were found in lane 2, but the intensity of the 18 kDa band was greater in this boiled sample (lane 2) than in the non-boiled sample (lane 1). This difference in intensity could be due to the exposure of hidden epitopes after complete denaturation of the peptide by boiling. Under the boiled and reduced conditions, CT separated into a reduced CTA1 domain (21.8 kDa) and a CTB monomer, as shown in lane 6. On the other hand, neither the 28 kDa nor the18 kDa band detected in the OMV sample was reduced (lane 3). Both bands migrated in the same manner as under non-reduced conditions (lane 2). These results indicate that the CT encapsulated within 569B OMVs isolated from a nutrient broth culture consists of two polypeptides of CT but no intact CT holotoxin. Because we have previously shown that the OMV-associated CT is biologically active and induces the same morphological response as intact CT [18], we hypothesized that at least one of these polypeptides contains an active domain of CTA.

### 2.3. The CTB-Subunit Is Not Associated with 569B OMV-Encapsulated CT

**Figure 3:**
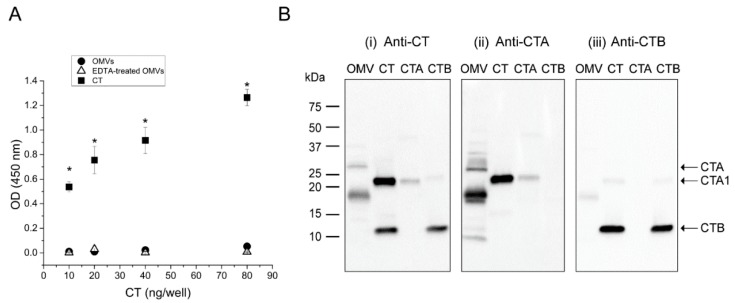
Composition analyses of *V. cholerae* 569B OMV-associated CT. (**A**) GM1 ELISA of intact (circles) and EDTA-treated (triangles) 569B OMVs using purified CT as a standard (squares). Neither intact nor EDTA-treated OMVs bound to GM1. Data are expressed as mean ± SD (*n* = 3). One-way analysis of variance followed by Bonferroni’s multiple comparison test was used to determine the level of significance between samples. No significant difference was found between untreated and EDTA-treated OMVs, and a statistically significant difference was observed between purified CT and both intact and EDTA-treated OMVs: 12 comparisons; * *p* < 0.05/12 = 0.004. (**B**) Western blot analyses of *V. cholerae* 569B OMVs in the presence of purified CT, enzymatic A-subunit (CTA), and CTB (10 ng·µL^−1^) as standards under boiled and reduced conditions using polyclonal antibodies against (i) CT, (ii) CTA, and (iii) CTB. The two anti-CT reactive bands found in the OMVs were detected by the anti-CTA polyclonal antibody but were not recognized by the anti-CTB antibody.

To investigate the identity of the two anti-CT reactive peptides detected within the OMVs, a GM1 ELISA assay was performed using an anti-CT polyclonal antibody. Because only the B-subunit of the intact holotoxin is able to bind GM1 [11], we used this assay to quantify the presence of B-subunit in the OMV-associated CT. We have previously demonstrated that when *V. cholerae* 569B is cultured in a low-salt nutrient broth medium, CT is located exclusively inside the OMVs and therefore the vesicles do not bind GM1 [18]. Here, we reasoned that if the OMVs contain any CTB, we would observe binding of disrupted OMVs to the GM1-coated plate. For this reason, EDTA-disrupted and intact vesicles were added to the wells of a GM1-coated plate in serial dilution. A serial dilution of CT was also used as the positive control. As expected, a dose-dependent increase in antibody binding was observed for the soluble CT, indicating binding of the toxin to the GM1. However, neither intact nor EDTA-disrupted OMVs demonstrated any binding to GM1, suggesting the absence of CTB in the vesicles (Figure 3A).

In addition, the 569B OMVs were analyzed by Western blot under boiled and reduced conditions to compare the binding of anti-CT, anti-CTA and anti-CTB antibodies to the CT fragments found in the OMVs. The same volume of CT, CTA, and CTB (10 ng·μL^−1^) was also loaded in all gels as controls. In Figure 3B, i, the anti-CT antibody identified two bands in the OMVs, as described previously. This antibody also recognized a band corresponding to the molecular weight of CTA1 in the CT and CTA samples, as well as a band consistent with CTB monomer in the CT and CTB samples. As shown in Figure 3B, ii, the anti-CTA antibody recognized both of the bands detected in the OMVs by the anti-CT antibody, in addition to two faint bands with molecular weights of approximately 32 kDa and 8 kDa. Because these two bands were not detected by the anti-CT antibody, we expect that they are cross-reacting bands. This antibody also detected the CTA1 band in the CTA and CT samples, similarly to the anti-CT antibody, and did not recognize the CTB band in the CT and CTB samples. Additionally, in Figure 3B, iii, the anti-CTB antibody strongly associated with the CTB monomer in the CT and CTB lanes. This antibody also slightly recognized the 18 kDa band in the OMV sample as well as the CTA1 in the CT sample. Because these two bands have been detected strongly by the anti-CTA antibody, we expect that this weak binding is a cross-reaction due to the high concentration of these two bands. Together, these results demonstrate the absence of CTB within the *V. cholerae* 569B vesicles isolated from a low-salt nutrient broth culture and the presence of two anti-CTA reactive polypeptides of CT with molecular weights of 28 kDa and 18 kDa. Because the 28 kDa peptide was non-reducible, we expect that it is an un-nicked form of CTA. Additionally, because the epitope of the anti-CTA antibody resides in the CTA1 subunit [23,24], we propose that this 18 kDa peptide is a fragment of CTA1. For the sake of brevity, we denote this fragment “CTA*” in the remainder of the paper.

### 2.4. OMVs Encapsulate CTA from the Periplasmic Pool

**Figure 4:**
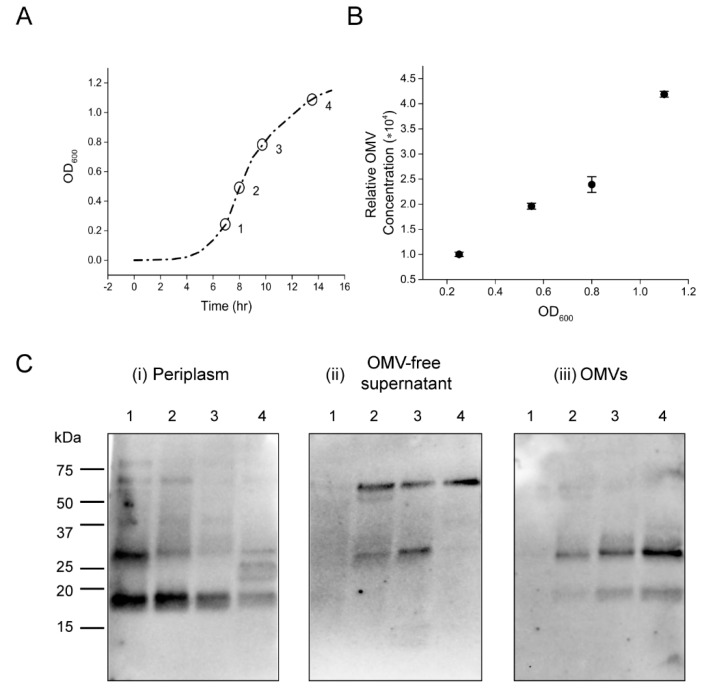
Characterization of CT assembly and secretion in *V. cholerae* 569B. (**A**) Growth curve of *V. cholerae* 569B. Four cultures were grown to an OD_600_ of 0.25, 0.55, 0.8, and 1.1. Selected time points are marked by circles on the growth curve. The line is a guide to the eye. (**B**) The relative concentration of isolated OMVs produced during the *V. cholerae* 569B growth cycle. The quantity of OMVs was found to increase linearly as measured by the fluorescence of the lipophilic dye, FM 4-64. Data are shown as mean ± SD (*n* = 3). (**C**) Western blot analyses of (**i**) periplasmic, (**ii**) OMV-free supernatant, and (**iii**) OMV fractions extracted from four *V. cholerae* 569B cultures, analyzed under non-boiled and non-reduced conditions, using a polyclonal anti-CT antibody. Lanes 1 to 4 represent samples with OD_600_ of 0.25, 0.55, 0.8, and 1.1, respectively.

We next wanted to characterize the CT composition of OMVs formed at different growth phases to follow the two anti-CTA reactive peptides as they are encapsulated within the vesicles. We hypothesized that these peptides might be excess CTA-subunits remaining after the holotoxin (CTAB5) assembly reaction in the periplasm, which become entrapped in OMVs. To address these questions, periplasm, OMVs, and OMV-free supernatant fractions of four *V. cholerae* 569B samples cultured in nutrient broth collected from early to late exponential phases (OD_600_ of 0.25, 0.55, 0.8, and 1.1) were extracted. Figure 4A demonstrates the growth curve of the bacteria and the selected time points. Initially, the quantity of OMVs produced during all phases was quantified by the fluorescence of the lipophilic dye, FM 4-64, and was found to increase linearly during the growth of the bacteria, demonstrating that OMV secretion is not limited to a specific phase of growth (Figure 4B).

Western blot analyses were performed on all of the samples from the three fractions using a polyclonal anti-CT antibody. Due to the heat-sensitivity of the CTB5 pentamer (Figure 2), all analyses were carried out under non-boiled and non-reduced conditions to monitor the assembly and oligomerization of the subunits and secretion of CT. Samples from the periplasmic fractions were normalized by their OD_600_; therefore, each sample represents the periplasm of an average bacterium in each phase. As shown in Figure 4C, i, two light bands (84 kDa and 68 kDa) along with two major bands (28 kDa and 18 kDa) were found in the periplasm from the early log phase (lane 1) with a decrease in intensity during the later phases (lanes 2 to 4). These bands correspond to fully assembled CT, near-assembled oligomer, CTA, and CTA*, respectively. The weak intensity of the fully assembled CT and near-assembled oligomer in the periplasm supports the reported minimal production of CT in low-salt culture [21,22].

In the supernatant fraction (Figure 4C, ii), two bands with molecular weights of 56 kDa and 28 kDa, corresponding to CTB5 pentamer and CTA, respectively, were observed from the early log phase (lane 2) with a slight increase in intensity over the growth cycle. However, the CTA* band (18 kDa) we have observed in the periplasm and OMVs was absent in this fraction. The two observed bands are consistent with those shown for CT in Figure 2 (lane 4); therefore, these findings confirm the extracellular secretion of assembled periplasmic CT through the T2SS. Additionally, in the OMV fraction (Figure 4C, iii), the CTA and CTA* polypeptides were detected with a constant increase in intensity from the early to late logarithmic phases. We reasoned that the weak intensity of the CTA* in the OMV fraction in comparison to its strong intensity in the periplasmic fraction is due to the inaccessibility of the epitope(s) of the non-boiled CTA^*^ in its native conformation within OMVs, as previously shown in lanes 1 and 2 of Figure 2. This weak intensity could be also due to the degradation of the CTA* within the periplasm. As observed previously, the CTB5 pentamer was not detected in the OMV fraction, confirming that in a low-salt medium, CT holotoxin is not secreted in association with OMVs. These results demonstrate that encapsulation of CTA into the OMV lumen occurs continuously in the form of two 18 kDa and 28 kDa peptides in a process that appears to occur alongside the secretion of CTAB5 holotoxin via the T2SS.

### 2.5. The Encapsulation of CTA in OMVs Is Conserved among OMVs from Several O1 Inaba Strains

**Figure 5:**
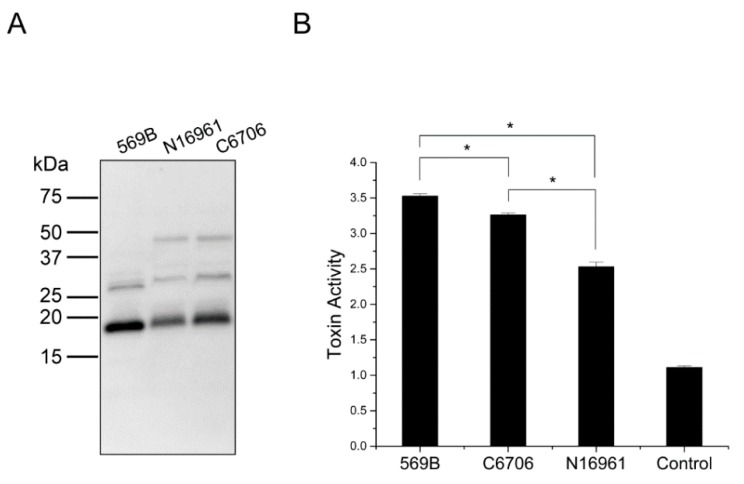
Characterization of *V. cholerae* O1 Inaba OMVs. (**A**) Western blot analyses of OMVs from 569B, N16961, and C6706 strains under boiled and reduced conditions. Similar to 569B OMVs, intact CT was absent in N16961 and C6706 OMVs. Both anti-CT reactive bands that were detected in 569B OMVs were found in N16961 and C6706 OMVs. (**B**) Quantitative analysis of OMV-mediated cytotoxicity. FHs 74 intestinal epithelial cells were treated with isolated OMVs from the 569B, N16961, and C6706 strains. Control cells were incubated with Hank’s balanced salt solution. Based on the percentage of cell rounding, the change in cell morphology was scored on a scale of 1 (spindly) to 4 (rounded). Data are presented as mean ± SD (*n* = 3). The level of significance was determined using one-way analysis of variance followed by Bonferroni post-hoc test: 3 comparisons; * *p* < 0.05/3 = 0.0167.

While Classical *V. cholerae* O1 strains can express CT under the standard in vitro conditions, El Tor strains are reported to be unable to induce virulence under these conditions and require more complex growth conditions termed AKI. The AKI conditions, which include two steps of static growth in a tube followed by growth in a shaking flask, do not mimic the physiological conditions in the small intestine [25]. Therefore, the in vivo virulence induction of the El Tor biotype is not well understood. We hypothesized that similar to the vesicles from Classical 569B strain, OMVs from El Tor strains might encapsulate CTA within OMVs through this mechanism even under so-called “non-virulence inducing” conditions. To investigate if our findings on CTA-association with O1 Classical Inaba 569B OMVs can be generalized to OMVs from O1 El Tor Inaba strains, we analyzed N16961 and C6706 OMVs. Cultures were grown in nutrient broth to an OD_600_ of 1.0, and OMVs were purified from each culture. Western blot analysis was performed on the vesicles under boiled and reduced conditions using an anti-CT polyclonal antibody (Figure 5A). 569B OMVs were used as the control. Similar to 569B vesicles, both CTA and CTA* bands were detected in N16961 and C6706 OMVs. The intensity of the bands was detected to be stronger in C6706 OMVs than in N16961 OMVs. Moreover, the signal detected for CTA* in 569B OMVs was stronger than the signals detected in the other two vesicles. A band between 37 kDa and 50 kDa markers was also detected in both N16961 and C6706 vesicles which was absent in 569B OMVs. This result suggests that the encapsulation of CTA and CTA^*^ (and not AB5 CT) is conserved among studied Inaba O1 *V. cholerae* strains.

We next investigated the biological activity of OMVs purified from the N16961 and C6706 strains using a cell morphology scoring assay. We have previously shown, using this assay, that similar to soluble CT, the 569B OMVs induce an alteration in the morphology of the host cells [18]. Therefore, to compare the cytotoxicity of N16961 and C6706 OMVs with 569B OMVs and investigate whether they induce a similar cellular response, FHs 74 Int cells were treated with vesicles from each strain for 3 h. The resulting morphology was scored on a scale of one to four, with one being elongated and four being rounded. Similar to 569B OMVs, cell rounding was observed for samples treated with both N16961 and C6706 OMVs (Figure 5B). The C6706 vesicles, which had a higher concentration of both CTA and CTA* than the N16961 vesicles, induced a greater cellular response. Additionally, the cytotoxicity score of 569B vesicles was greater than that of the other two strains.

### 2.6. The Encapsulation of CTA in OMVs Increases with an Increase in the Culture Osmolarity

**Figure 6:**
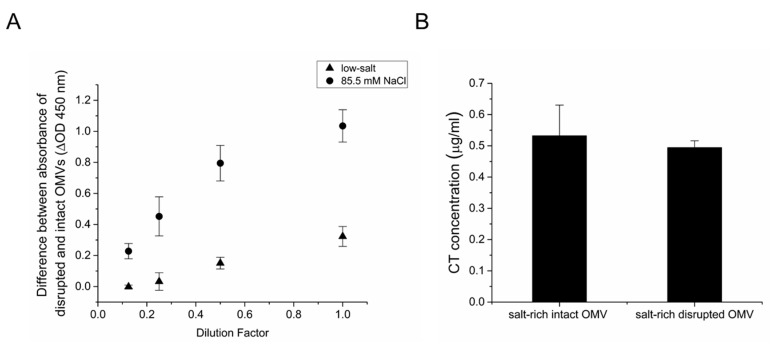
The effect of osmolarity on encapsulation of CT subunits with OMVs. (**A**) Comparison of CTA encapsulation in 569B OMVs from cultures grown in either low-salt (triangles) or 85.5 mM NaCl-containing growth medium (squares). Indirect noncompetitive ELISA was performed for intact and EDTA-disrupted 569B OMVs, and the difference between the binding to anti-CTA polyclonal antibody was calculated to measure the intravesicular CTA signal. More CTA was detected in the lumen of vesicles isolated from salt-rich medium than in the lumen of vesicles isolated from low-salt medium. Data are expressed as mean ± SD (*n* = 3). (**B**) GM1 ELISA of intact and EDTA-disrupted 569B OMVs under salt-rich conditions using purified CT as a standard. No increase in the concentration of CTB was found for EDTA-treated OMVs. Data are presented as mean ± SD (*n* = 3). No significant difference was observed between the two samples as determined by an unpaired two-tailed t test.

Together, these results demonstrate that CTA is encapsulated within the OMVs under low-salt conditions. Additionally, we demonstrated that under salt-rich conditions, CT is associated with the surface of OMVs. To determine if the culture osmolarity affects the encapsulation of CTA within OMVs, an indirect ELISA assay was performed using an anti-CTA polyclonal antibody, as described previously [18]. In this assay, an ELISA plate was coated with a serial dilution of either intact or EDTA-disrupted OMVs from low-salt and salt-rich cultures. To quantify the intravesicular CTA concentration, the difference between the signals detected for the disrupted and intact vesicles was calculated for each condition. As shown in Figure 6A, the signal increases in a dose-dependent manner for both OMVs grown in low-salt and salt-rich conditions, indicating that CTA is present within the lumen of the OMVs. A greater dose-dependent signal was detected for OMVs grown under salt-rich conditions, suggesting that an increase in the osmolarity of the culture leads to greater encapsulation of CTA fragments within the vesicles in addition to the observed increase in CT association with the surface of the vesicles (Figure 1).

Next, we wanted to investigate whether CTB is encapsulated within the vesicles under salt-rich culture conditions. For this reason, a GM1 ELISA was performed using an anti-CT antibody to compare the CTB concentration in intact and EDTA-disrupted OMVs from a salt-rich culture. A serial dilution of CT was also used as a standard. Figure 6B demonstrates that, as expected, CTB is associated with the surface of the intact vesicles. However, no increase in CTB concentration was detected in the disrupted OMVs. Together, these results indicate that the encapsulation of CTA within the vesicles increases with an increase in osmolarity of the growth medium. However, under neither low-salt nor salt-rich conditions is CTB encapsulated within the OMVs.

## 3. Discussion

It has been previously reported that Classical biotype strains of *V. cholerae* O1 produce CT in a NaCl-dependent manner [21]. In this work, we first demonstrated that the addition of salt to the culture not only increases the secretion of CT in free form but also results in the localization of extracellularly secreted CT holotoxin (AB5) on the surface of OMVs as well as encapsulated within the OMV. We then further characterized the structure and mechanism of encapsulation of CT in *V. cholerae* OMVs. To specifically characterize the lumenal CT and avoid contamination of vesicles with surface-associated CT, we performed our studies under low-salt culture conditions. We demonstrated that 569B OMV-encapsulated CT lacks a mature AB5 multimer structure and instead contains two anti-CTA reactive polypeptides. By analyzing the periplasm of *V. cholerae*, we found that the two anti-CTA reactive polypeptides that we observe inside the 569B OMVs constitute a major fraction of the periplasmic CT. Together, the results in this paper suggest that a fraction of the periplasmic CTA-subunit does not participate in the CT holotoxin assembly process and is instead continuously encapsulated within OMVs. We propose that this OMV-association comprises an important mechanism of delivery of the active CTA-subunit that requires neither the CTB-subunits nor GM1.

Heat-labile enterotoxin (LT) is another AB5 toxin, closely related to CT, which is produced by enterotoxigenic *Escherichia coli* (ETEC). Previous studies on the secretion of LT from *V. cholerae* have demonstrated that LT subunits enter the periplasm as monomers and spontaneously oligomerize into the assembled toxin before they travel across the outer membrane [26]. Additionally, in vitro studies of CT have shown that CTA is capable of binding to B-subunits that are in the assembly process but not to the fully assembled CTB5, suggesting that in the periplasmic space, CTA coordinates the CT holotoxin assembly by association with the intermediates in B-pentamer assembly [27]. In agreement with these findings, our results reported here indicate that under low osmolarity conditions, native and near-native CT in the periplasm is secreted as mature (AB5) CT into the supernatant during the early logarithmic phase. However, these forms of toxin constitute only a small fraction of the periplasmic CT. We found, during all growth phases, two major anti-CTA reactive polypeptides in the periplasm that were not bound to the oligomeric forms of CTB and instead were continuously secreted through OMVs.

In spite of numerous studies on the wide range of exoproteins recognized by the T2SS, little is known about the secretion signal that mediates the translocation of proteins across the outer membrane [28]. Studies about LT secretion from *V. cholerae* have shown that the A-subunit can accelerate the B-subunit pentamerization in the periplasm [27], while in the absence of A-subunit, the B-subunit is still able to be assembled into a stable pentamer and be secreted through the T2SS apparatus. However, in the absence of the B-subunit, the A-subunit has not been found to be secreted into the medium, suggesting that the secretion signal of LT resides in the B-subunit [29,30]. Recent electron microscopy analyses also confirmed that CTB5 can bind to the entrance of the periplasmic vestibule of the secretin GspD, the major T2SS outer membrane protein in *V. cholerae* [31,32]. On the other hand, Connell et al. have proposed that for both LT and CT, the transport signal is instead a conformational motif within the native or near-native structure of the secreted toxin [33].

Our finding of two assembled and near-assembled oligomers in the periplasm, which are later secreted into the supernatant as CTAB5, is in agreement with previous studies that the CT secretion signal resides in the B-pentamer. Furthermore, the continuous encapsulation of the 28 kDa periplasmic CTA in OMVs, especially after the late logarithmic phase, where all of the CT and near CT bands are absent in the periplasm and no more CT is secreted in supernatant, indicates that in the absence of the CTB-pentamer, CTA lacks a secretion signal to be translocated individually across the outer membrane. Instead, CTA along with the periplasmic CTA* appears to be constantly packaged into OMVs and secreted through this pathway. Given that a majority of periplasmic CTA is secreted through OMVs, it is conceivable that CTA contains a signaling motif that enables packaging within the budding OMV through a secondary mechanism.

In laboratory-grown cultures of *V. cholerae*, secreted CT normally becomes enzymatically activated by extracellular proteases, which cleave CTA at the nick site, Arg-192 [5]. Hemagglutinin/protease (HAP), VesA, and VesB are three proteases secreted through the T2SS that have been demonstrated to contribute to proteolytic cleavage of secreted CT; inhibition of these enzymes has been demonstrated to result in the secretion of un-nicked CT [34,35]. However, Lencer et al. have shown that endogenous proteases of the host cell are able to activate endocytosed CT, and processing of the A-subunit by *V. cholerae* proteases prior to its internalization in host cells may not be essential in vivo [36]. In light of these findings, we suggest that the reason that the OMV-associated 28 kDa, anti-CTA reactive polypeptide is unable to be reduced to CTA1 under boiled and reduced conditions is that the CTA packaged within the vesicles is un-nicked, possibly due to the absence of bacterial proteases in the periplasmic space that are able to cleave CTA at residue Arg-192.

In addition, we detected an 18 kDa, non-reducible, anti-CTA reactive peptide (CTA*) in both the periplasm and vesicles of 569B strain, which, similar to CTA, was conserved among the studied El Tor and Classical strains of *V. cholerae*. We reasoned that because the epitope recognized by the anti-CTA antibody resides within the CTA1 subunit [23], the CTA^*^ is a fragment of CTA1. At this point, it is not clear how CTA degrades to form these CTA1 fragments. One possible mechanism could be the partial cleavage of periplasmic CTA by unknown periplasmic proteases, which nick CTA at an alternative site.

We have previously shown that CT does not have any affinity for *V. cholerae* 569B LPS isolated from a low-osmolality nutrient media. Thus, it does not bind to the outer surface of the OMVs after secretion [18]. Our results here, however, demonstrate that under high osmolarity conditions, extracellularly secreted CT binds to the surface of OMVs. It has been reported that salt stress may alter the bacterial LPS structure [37,38]. We therefore propose that an increase in the salt level of the growth medium could change the structure of the *V. cholerae* LPS, and the interaction of CT with this modified LPS might lead to association of CT with the surface of vesicles.

A number of studies have demonstrated the association of various virulence factors with OMVs either on their outer surface or within their lumen. In ETEC, the OMV-associated LT has been found to partially reside inside the OMVs, while another fraction of the toxin is located on the surface of the OMVs [39]. Horstman et al. have demonstrated that the partial association of the toxin with the outer surface of OMVs is due to the LT-LPS interaction upon secretion of LT through the T2SS [40]. Moreover, an early study on periplasmic LT has shown that only about 50% of the periplasmic pool of free LTA associates with LTB to form LT holotoxin [41]. Based on these studies and our findings, we propose that the intravesicular LT, similar to CT, is mainly composed of the A-subunit and that *V. cholerae* and ETEC share a similar process in packaging the biologically active periplasmic A-subunit within their vesicles.

**Figure 7:**
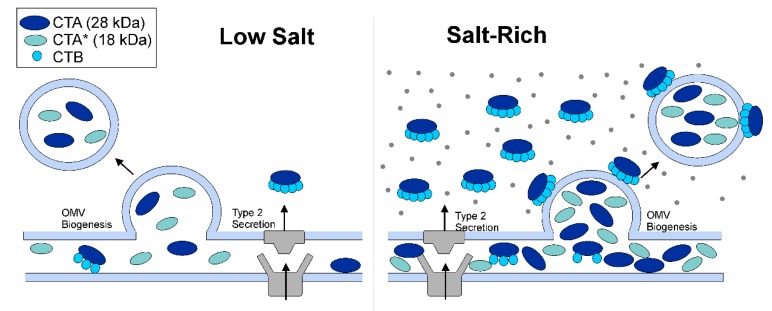
Schematic of proposed mechanism of CTA and CT holotoxin assembly and secretion via T2SS and OMVs under low-salt and salt-rich conditions. In this schematic, CTB is shown as turquoise circles, CTA (28 kDa) as dark blue ovals, and CTA* (18 kDa) as light blue ovals. Our results demonstrate that an increase in the osmolarity of the culture results in an increase in the secretion of CT through both T2SS and OMVs. We propose that three forms of CT can be found under salt-rich conditions; (1) the free, soluble CT, (2) the OMV-surface associated CT, and (3) the OMV-encapsulated CTA.

In summary, we propose that there are three forms of biologically active CT; the well-studied free CT that is secreted extracellularly, the OMV-surface associated CT, and the OMV-encapsulated form which only constitutes the CTA fragments (Figure 7). Our studies under low-osmolarity condition suggest that the latter does not require the CTB-GM1 interaction for endocytosis and only relies on OMVs for host-cell entry. Therefore, OMV-encapsulation might be a secondary strategy of the bacteria to deliver active CTA to host cells.

## 4. Materials and Methods

### 4.1. Chemicals

CT and monosialoganglioside GM1 were purchased from Sigma-Aldrich^®^ (St. Louis, MO, USA). Rabbit polyclonal antibody against CT (ab123129) and CTB (ab34992) were obtained from Abcam (Cambridge, UK). Rabbit polyclonal antibody against CTA (AB-43) was purchased from Advanced Targeting Systems (San Diego, CA, USA).

### 4.2. Strains and Culturing Conditions

*V. cholerae* O1 Classical Inaba 569B (ATCC^®^ 25870™, Manassas, VA, USA) as well as El Tor Inaba strains C6706 and N16961 (provided by Dr. John Mekalanos) were used in this study. Cultures were grown in Difco nutrient broth (BD, Sparks, MD, USA), unless otherwise stated in the text (all at 37 °C with aeration).

Human fetal small intestinal FHs 74 cells (ATCC^®^ CCL241™, Manassas, VA, USA) were grown in Hybri-care medium (ATCC 46-X™, Manassas, VA, USA). The medium was supplemented with 10% fetal bovine serum (Sigma-Aldrich^®^, St. Louis, MO, USA) and 30 ng·mL^−1^ epidermal growth factor (Sigma-Aldrich^®^, St. Louis, MO, USA) at 37 °C, with 5% CO_2_. The medium was changed every two to three days.

### 4.3. Vesicle Purification

OMVs from cultures of 569B, N16961, and C6706 strains of *V. cholerae* (OD_600_ of 1.0) were isolated as described [18] and resuspended in the same volume of PBS. The samples were stored at 4 °C and used within a week.

### 4.4. GM1 ELISA

In the osmolarity study, a GM1 ELISA assay was performed as described previously [18] to quantify the concentration of CT in each sample using an anti-CT polyclonal antibody. Briefly, 569B strain was cultured in either standard or 85.5 mM NaCl-supplemented nutrient broth until OD_600_ of 1.0. Subsequently, the OMVs and OMV-free supernatant fractions were isolated from each culture. A GM1-coated plate was treated with 100 µl of each sample in triplicate in the presence of a serial dilution of purified CT with known concentration as a standard. The concentration of CT in each sample was measured accordingly.

A GM1 ELISA assay was performed to analyze the presence of CTB inside the vesicles. To expose the intravesicular CT, 569B OMVs were lysed with 0.1 M EDTA for 2 h at 37 °C. A serial dilution of CT, intact OMVs, and EDTA-treated 569B OMVs with CT concentrations ranging from 80 ng per well to 10 ng per well was prepared. After blocking the GM1 pre-coated wells of Maxisorp (Nunc, Thermo Fisher Scientific, Waltham, MA, USA) 96-well polystyrene microtiter plates with 3% (*w*/*v*) fatty acid-free BSA, 100 µL of each sample was added to the wells in triplicate (2 h, room temperature). CT was then detected using an anti-CT polyclonal antibody (1:5000) and secondary antibody, followed by the development of the CT-antibody complex using 3,3’,5,5’-tetramethylbenzidine (TMB) substrate solution (Thermo Fisher Scientific, Waltham, MA, USA).

### 4.5. Western Blot Analysis

Samples were separated by SDS-PAGE on an 8–16% mini-protean TGX precast protein gel (BioRad, Hercules, CA, USA) under the conditions described in each section. The proteins were then transferred to a nitrocellulose membrane for 1.5 h at 45 V, blocked with 5% non-fat dry milk and incubated with the primary and secondary antibody (goat anti-rabbit immunoglobulin G conjugated to horseradish peroxidase (HRP), 1:5000, Southern Biotech, Birmingham, AL, USA). Subsequently, signals were developed with an enhanced chemiluminescence (ECL) reagent kit (Thermo Fisher Scientific, Waltham, MA, USA).

### 4.6. Isolation of Periplasm, OMVs, and OMV-Free Supernatant

To investigate the CT assembly and secretion process, four 100 mL cultures of the 569B strain were grown to selected time points on the growth curve. The OD_600_ of the cultures were 0.25 (early logarithmic phase), 0.55 (mid logarithmic phase), 0.8 (late logarithmic phase), and 1.1 (early stationary phase). Bacteria were harvested at each time point by centrifugation (3000× *g*, 8 min, 4 °C), and the supernatants were saved for later analyses. The periplasm was extracted from the bacteria as described before [42]. Briefly, bacterial pellets from the cultures with OD_600_ of 0.25, 0.55, 0.8, and 1.1 were resuspended in 0.5, 1.1, 1.6, and 2.2 mL of TSE buffer (200 mM Tris-HCl, pH 8.0, 500 mM sucrose, 1 mM EDTA), respectively. The samples were incubated on ice for 30 min followed by a centrifugation step (16000× *g*, 30 min, 4 °C). The supernatants containing the periplasmic extracts were then collected. Samples were separated by SDS-PAGE under boiled and reduced conditions, followed by immunoblot analysis with a polyclonal antibody against CT. The loaded samples were normalized relative to their OD_600_ to represent the concentration of CT in the periplasm of an average bacterium at each growth phase.

The 596B vesicles were isolated from the OMV-free supernatant of samples at various optical densities using ultracentrifugation, as described previously [18]. The vesicle pellet was resuspended in the same volume of PBS. The relative concentration of OMVs at each point on the growth curve was determined using the lipophilic dye, FM 4–64 (5 µg·mL^−1^; Invitrogen, Carlsbad, CA, USA). The fluorescence (λ_excitation_ = 512 nm, λ_emission_ = 620 nm) of each sample was measured on a microplate reader. The OMV and OMV-free supernatant samples were then separated by SDS-PAGE under boiled and reduced conditions and were subjected to Western blot analysis using a polyclonal anti-CT antibody.

### 4.7. Cytotoxicity Assay

To compare the cytotoxicity of vesicles isolated from 569B, N16961, and C6706 strains, a cell morphology scoring assay was performed as described before [18]. Briefly, 4 × 10^4^ of FHs 74 Intestinal epithelial cells were grown overnight on a 96-well tissue culture treated plate (CELLSTAR^®^, Greiner Bio-One, Frickenhausen, Germany). Following three washes with Hank’s balanced salt solution (HBSS) buffer, cells were treated in triplicate with OMVs from each strain diluted in HBSS for 3 h. Cells treated with HBSS were used as a control. The cells were imaged using a Nikon Eclipse TE2000-U inverted microscope (Nikon Instruments Inc., Melville, NY, USA) at 20× magnification. The OMV-mediated cytotoxicity was then scored based on the percentage of cell rounding.

### 4.8. Indirect ELISA

To investigate the effect of a change in the osmolarity of the 569B culture on the concentration of CTA in the lumen of OMVs, an indirect noncompetitive ELISA was performed using an anti-CTA polyclonal antibody, as described previously [18]. Briefly, 569B cultures were grown in nutrient broth in the absence or presence of 85.5 mM NaCl and OMVs were extracted from each culture, accordingly. An ELISA plate was then treated with 100 μL of serially diluted samples of either intact or EDTA-disrupted OMVs from each condition followed by incubation with the primary and secondary antibody. To compare the intravesicular CTA cargo of the OMVs in low-salt and high-salt conditions, the signals detected for intact and EDTA-disrupted vesicles were subtracted.

### 4.9. Statistical Analysis

Statistical analysis was performed using one-way analysis of variance, followed by the Bonferroni test for multiple comparisons.
